# Prevalence of functional difficulty among school-aged children and effect on school enrolment in rural southern India: A cross-sectional analysis

**DOI:** 10.21203/rs.3.rs-4154190/v1

**Published:** 2024-04-22

**Authors:** Bobeena Rachel Chandy, Calum Davey, William E Oswald, Saravanakumar Puthupalayam Kaliappan, Kumudha Aruldas, Lena Morgon Banks, Smitha Jasper, Guru Nagarajan, Sean Galagan, David S Kennedy, Judd L Walson, Beena Koshy, Sitara SR Ajjampur, Hannah Kuper

**Affiliations:** Christian Medical College; National Institute of Teaching; RTI International; Christian Medical College; Christian Medical College; London School of Hygiene & Tropical Medicine; Christian Medical College; Christian Medical College; University of Washington; London School of Hygiene & Tropical Medicine; University of Washington; Christian Medical College; Christian Medical College; London School of Hygiene & Tropical Medicine

**Keywords:** functional difficulty, disability, school enrolment, effect modification, India

## Abstract

Despite the large number of children in India, there is little information on the impact of children’s disability on school enrolment, and how this differs by population. We estimated the prevalence of childhood disability in two sites in Tamil Nadu, southern India, and the effect of functional difficulty on school enrolment. We used a parent-reported survey containing the UNICEF-Washington Group questions to identify children aged 5 to 17 years with functional difficulty during a census conducted for an ongoing trial. We estimated pooled- and gender-specific prevalence of functional difficulty among 29,044 children. We fitted regression models to identify subgroups with higher rates of functional difficulty and the effect of functional difficulty on reported school enrolment. We estimated the modification of the effect of functional difficulty by age, gender, socioeconomic status, household education, and sub-site, on additive and multiplicative scales. We found of 29,044 children, 299 (1.0%) had any functional difficulty, equal among boys and girls. Being understood (0.5%) and walking (0.4%) were the most common difficulties. Functional difficulty was strongly associated with non-enrolment in school (Prevalence ratio [PR] 4.59, 95% CI: 3.87, 5.43) after adjusting for age, gender, and site. We show scale-dependent differences between age and socioeconomic groups in the effect of functional difficulty on enrolment. This study shows that at least one in a hundred children in this region have severe functional difficulties and nearly half of these children are not enrolled in school, highlighting the need for further efforts and evidence-based interventions to increase school enrolment among these groups.

## Introduction

Childhood disability continues to be a low priority on the global development and health agenda [1], despite the large and growing number of children affected, and the fact that the period of childhood and adolescence influences the entire life course [2]. The 2004 Global Burden of Disease Study estimated that 93 million or 5.1% of children aged 0–14 years had moderate/severe disability, of whom 13 million (0.7%) had severe disability [3, 4]. More recent work has put these numbers far higher. For instance, the 2017 Global Burden of Disease Study estimated that there were 291 million (11.2%) children and adolescents with one of four specified “disabilities” (epilepsy, intellectual disability, vision or hearing loss)(5). This figure grows further if more conditions are included, which includes various impediments to functioning [1, 6]. Not only is the number of children with disabilities large, but new research shows that it is rising as a result of population growth [1]. However, uncertainty remains about these figures due to variation in methods used to assess childhood disability, resulting in varying and non-comparable estimates. The UNICEF-Washington Group Child Functioning Module was introduced in 2017 to standardise the assessment of disability in children aged 2–17 years [7]. Its widespread use should increase the amount and comparability of international estimates, but to date few publications are available that have used this tool.

No matter how disability is measured, children who are found to have disabilities are left behind in different domains of life, including in education. A 2018 UNESCO report demonstrated through data from 49 countries that people with disabilities had consistently worse educational outcomes compared to their peers without disabilities, whether measured in terms of school enrolment, school completion, mean years of schooling, or literacy levels [8]. Data from 6 countries also showed that out-of-school rate was higher among children with disabilities than children without in both primary school (34.5% versus 14.1%) and secondary school (25.7% versus 17.5%). Lower levels of education of children with disabilities were also shown in the 2011 World Report on Disability [3] and the 2018 Flagship Report on Disability and Development [9]. For example, having a disability reduced the probability of attending school by 30.9 percentage points in an analysis across 15 low and middle income countries [10].

The exclusion of children with disabilities from education is a violation of their rights, as set out by the UN Convention on the Rights of the Child (articles 23, 28 and 29) [11], and the UN Convention on the Rights of Persons with Disabilities (article 24) [12]. It will also make it more challenging to reach SDG 4 on “inclusive and equitable quality education,” which includes specific targets on eliminating gender disparity and discrimination against people with disabilities, among other priority groups [13]. Different rights and goals are interlinked, and it will be more difficult to achieve employment for all, as an example, without first reaching all with education.

Children with disability are not a homogenous group, and their level of inclusion in education may be affected by a range of factors, such as gender, poverty level and impairment type. However, there is a lack of evidence of predictors of which children with disability are going to school. This gap is important as evidence is needed on which children with disability are most likely to be excluded to plan interventions to promote educational inclusion. Furthermore, there is currently a lack of data on which interventions are effective for improving uptake, and so evidence is first needed on the key issues before appropriate solutions can be developed [14, 15]. The limited data that is available suggests that boys with disabilities may fare better than girls. The UNESCO data showed that boys with disabilities had almost one year more of schooling than girls with disabilities (5.4 versus 4.3), and men with disabilities had higher literacy rates than women with disabilities (62% versus 49%) [8]. Data from Plan International across 30 countries showed that children with certain impairment types were less likely to go to school, especially those with learning or communication impairments [16]. Generally, however, such data in relation to school attendance is lacking.

India is an important country to consider for disability and educational inclusion, given its size and global influence. The 2011 Indian Census, using a simple assessment of disability (“Is this person mentally/physically disabled?”) estimates that 2.2% of the population had disabilities, including 1.5% of children aged 5–9 years and 1.8% of children 10–19 years [17]. These prevalence estimates are likely to substantially underestimate the true levels, given the simplicity of the question used [18]. Subsequently, a large community study from five diverse Indian sites showed that pooled estimates of prevalence of neurodevelopmental disorders (visual impairment, epilepsy, cerebral palsy, hearing impairment, speech disorders and autism for all children, and attention deficit hyperactivity disorder and learning disorder among 6–9 year-olds) were 9.2% and 13.6% in children 2–6 years and 6–9 years of age, respectively [19]. India has established a comprehensive legal framework for inclusive education. The Right to Education (RTE) Act of 2009 confirmed the right of all children to free and compulsory education [20]. The Rights of Persons with Disabilities Act 2016 made the commitment that “every child with benchmark disability between the age of six to eighteen years shall have the right to free education in a neighbourhood school, or in a special school, of his choice” [21]. The National Education Policy 2020 went further still and made a range of commitments towards the inclusion of children with disabilities, such as appropriate training of teachers, appropriate learning material (e.g. Braille), accessible facilities and protecting the safety of children with disabilities [22]. Moreover, India has ratified both the UN Convention on the Rights of Person with Disabilities (2007) and the UN Convention on the Rights of the Child (1992), which protect the right to education for children with disabilities.

Barriers to accessing education remain, however, despite these commitments. As a result, school enrolment is far from complete. Estimates from the 2011 census show that 29% of all children aged 5–19 years do not attend school, but this rises to 39% of children with disabilities [17]. Enrolment was higher in primary than the higher levels of schooling and was better for children with certain types of impairment (hearing – 67%, seeing – 68%) than among children with intellectual impairments (47%), mental health conditions (34%) or multiple disabilities (37%). However, these estimates are now ten years out of date, and preceded some of the new legislation protecting the right to education of children with disabilities. Since 2011, the proportion of children in primary schools with disabilities has increased by 50% (0.8–1.2% of children enrolled in primary school in 2015/2016), although the proportion of children in secondary school with disabilities has remained unchanged (around 0.3%) [23]. This may represent improved recognition and reporting of disability, and these figures only capture children with official certifications of disability and do not capture children with disabilities out of school.

The aim of this study was to use UNICEF-Washington Group questions to estimate the prevalence of childhood disability in a population in Tamil Nadu, India and to estimate the effect of functional difficulty on school enrolment and examine how this effect may differ between sociodemographic groups.

## Methods

### Study design and participants

The DeWorm3 Project is a multi-country, cluster-randomised, controlled trial conducted in Benin, India, and Malawi to test feasibility of interrupting transmission of soil-transmitted helminths (STH) through three years of expanded mass drug administration (MDA) targeting all community members versus each of the current national STH MDA strategies. Each study site includes a population of at least 80,000 individuals, divided into 40 clusters of at least 1,650 individuals, considering administrative borders and geographic barriers. The trial in India has two sites in Tamil Nadu: the Timiri block in the Vellore Health Unit District (HUD) and villages in the Jawadhu Hills block of the Tiruvanamalai HUD. A census of all individuals residing within the study site is conducted yearly to enumerate the population [24, 25].

The current study took place in the India DeWorm3 site, during a trial population census update activity conducted between October 19, 2019 and February 3, 2020. At each household, a structured questionnaire was conducted with the head of household or an equivalent adult resident. The questionnaire collected information on household member demographics, school enrolment or highest education level, household ownership of key assets, and water and sanitation facilities. Age and gender were verified using state or central Government of India issued identification (*Aadhar* card, Electoral Identity Card, Driving License, or Birth Certificate). Enumerators observed the material of the floor, walls, and roof and collected Global Positioning System (GPS) coordinates at each household. All data were collected using an electronic questionnaire programmed using SurveyCTO software (Dobility, Inc; Cambridge, MA and Ahmedabad, India) on Android smartphones.

The information of all eligible individuals, aged 5 to 17 years and enumerated during the household census visit, was automatically sent to a second electronic questionnaire containing the UNICEF/Washington Group Module on child functioning and disability [7, 26]. This parent-reported survey module is designed to identify children with functional difficulties in population-based surveys. For most domains, it scores functional limitation on a scale comprising “no difficulty,” “some difficulty,” “a lot of difficulty,” and “cannot do.” Fieldworkers then conducted the questionnaire with the mothers or reported primary caregivers for each identified eligible child within the household.

### Outcomes

A child was classed as having functional difficulty if they reported “a lot of difficulty” or “cannot do” within the domains of seeing, hearing, walking, self-care, communication, learning, remembering, concentrating, accepting change, controlling behaviour, and making friends or reported “daily” anxiety, nervousness, worry or seeming sad or depressed.

During the census update activity, the respondent was asked whether each individual household member aged between 3 and 25 years attended school. Responses were then recorded as “not in education” or the specific level of education (Anganwadi centre; kindergarten; primary; middle; secondary; higher secondary; college; or ITI/diploma). For the current study, children were classed as not being enrolled in school if they were reported to be “not in education.”

### Covariates

Individual covariates included age in years, categorised as 5–9, 10–13, and 14–17 and male or female gender. Household covariates included: an indicator of household size dichotomised as < 5 or ≥ 5 from the number of residents; reported years in residence; education and marital status of the head of household; reported household caste, religion, and language. A measure of socioeconomic status was derived from a composite wealth index based on a principal component analysis (PCA) using various reported household assets (24). The wealth index was divided into five quintiles, and for the current analysis the lower 3 quintiles were classed as poorer and the upper 2 quintiles were classed as less poor.

### Statistical analysis

We estimated the prevalence of difficulty within each of the functional domains and in any of the domains in total and by gender. We then estimated prevalence ratios between levels of each of the candidate covariates and presence of any functional difficulty and, separately, reported school non-enrolment using modified Poisson regression [27].

For this, we fit generalised linear models, specifying a Poisson distribution and log link. We estimated robust standard errors at the household level to accommodate the binary outcomes and to account for potential outcome correlation between children for residence within the same household. We excluded individuals with missing outcome or covariate data (0.3%), and assumed these data were missing at random, that is, that the probability of having complete data is independent of the outcome after adjusting for included covariates. We report prevalence ratios with 95% confidence intervals as crude (bivariate) and adjusted for age category, gender, and site.

We examined heterogeneity of the effect of functional difficulty upon reported school non-enrolment with an aim towards identifying groups who may benefit from additional interventions to reduce school exclusion. We examined this effect modification by age dichotomised at the median (5–11 years versus 11–17 years), gender (male versus female), SES (higher versus lower), head of household education (any formal versus no formal education), and site (Timiri versus Jawadhu Hills). We estimated this effect modification on both multiplicative and additive scales, the latter of which is considered more meaningful from a public health perspective [28]. For the latter, we estimated the relative excess risk due to interaction (RERI) [28–30], using the interactionR package with confidence intervals calculated using the MOVER method [31]. When the exposures of interest have the same direction of effect upon the outcome, a RERI greater than 0 means greater than additive interaction or positivity, whereby we might observe a greater number of outcomes in the presence of the main effect and its modifier than we would expect based on the numbers of outcomes attributed to the effect and modifier separately. A RERI less than 0 means there is less than additive interaction or negative interaction. We adjusted for age, gender, and site in the interactions analysis.

All analyses were conducted using R version 4.0.3 (2022-04-29). De-identified data and analysis scripts are available upon request via LSHTM Data Compass.

## Results

**Figure 1:**
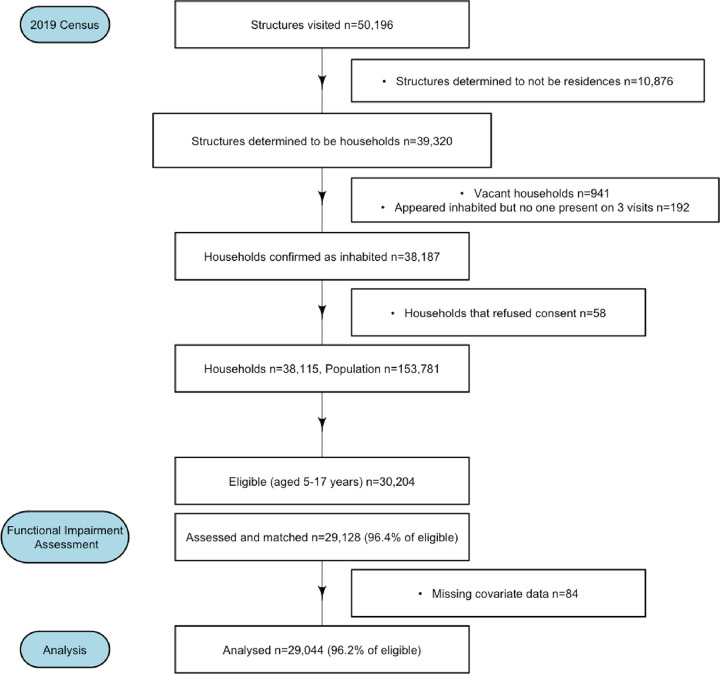
Flow chart of activities during the DeWorm3 census and assessment for functional difficulties in Tamil Nadu, 2019

A total of 29,044 children aged 5–17 years were included in the analysis, after excluding 84 with missing data (Fig. 1). The distribution of functional difficulties are shown in Table 1 by gender. Of 29,044 children, 299 (1.0%) had any functional difficulty. Few children had limitations with seeing, hearing, depression, or anxiety, while 0.3–0.4% of the children were reported to have limitations in the other domains, such as walking, remembering, or accepting change.

Few sociodemographic factors were associated with having any functional difficulty (Table 2). Many of the associations were close to the null before and after adjustment for age, gender, and site. There was some evidence of a positive association between any functional difficulty and living in the tribal Jawadhu Hills area as opposed to the rural Timiri area (PR: 1.30, 95% CI: 1.01 to 1.67), adjusting for age and gender. There was some evidence of a small positive association between any functional difficulty and household socioeconomic status, which was attenuated towards the null after adjusting for age, gender, and site.

**Figure 2:**
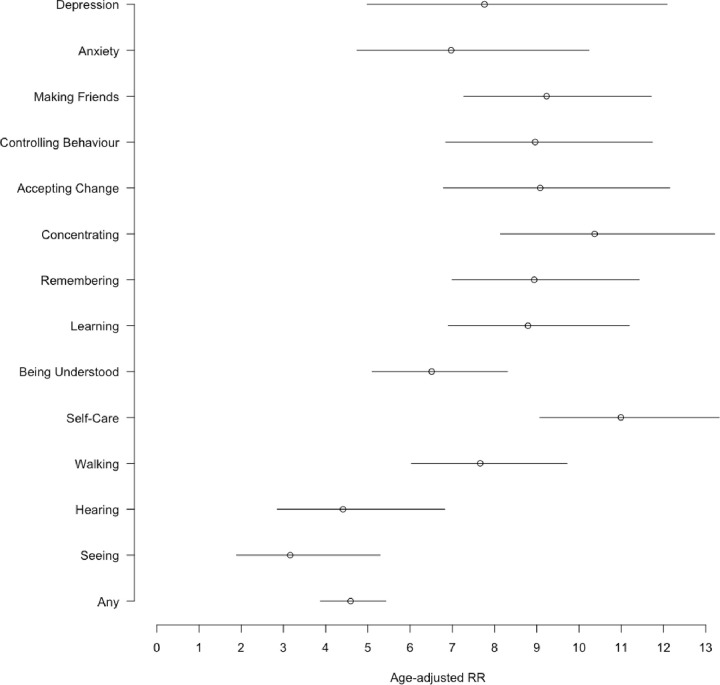
Age- and gender-adjusted associations (risk ratios) between domain-specific measures of functional impairment (as opposed to no impairment) and school non-enrolement.

Several of the analysed factors were associated with reported school non-enrolment (Table 3). Among children with functional difficulty, the prevalence of school non-enrolment (43.1%) was 4.59 times higher than that among children without functional difficulty (9.5%), after adjusting for age, gender, and sub-site (95% CI: 3.87 to 5.43). Prevalence of non-enrolment was higher in older age categories, and children aged over 14 years were 8.57 times more likely to be non-enrolled compared to younger children aged 5 to 9 years (95% CI: 7.60 to 9.66), adjusting for gender and site. Figure 2 shows the associations for each domain of the CFM, where the categories measured among older children are associated more strongly with non-enrolment. Gender was also found to be a contributor to non-enrolment, as girls were 16% more likely to not be enrolled as boys (PR: 0.84, 95% CI: 0.79 to 0.90). Prevalence of non-enrolment was observed to be higher in Jawadhu Hills compared to Timiri, after accounting for age and gender. However, after adjusting for area differences, children from poorer households, households in longer residence, or with less educated or unmarried household heads were found to have higher prevalence of non-enrolment.

The results of analysis of modification of the effect of any functional difficulty on school non-enrolment by selected factors is shown in Table 4 (domain-specific effects are shown in the **Web Appendix**).

In the case of age, the multiplicative and additive measures of effect modification differed in direction. In young children, prevalence of non-enrolment among those with any functional difficulty was nearly fourteen times that of children without functional difficulty (PR: 13.92, 95% CI: 9.11 to 21.27), while the same effect in older children was smaller (PR: 3.31, 95% CI: 2.54 to 4.30). Reflecting this, the age interaction measure on the multiplicative scale, equating the ratio of these age stratum-specific PRs, was 0.24 (95% CI: 0.14 to 0.39). The additive interaction measure of 2.03 in turn (95% CI: −6.43 to 9.97) was positive. This result reflects the greater joint association on the additive scale between older age and functional difficulty and not being enrolled in school (PR: 21.43, 95% CI: 15.95 to 28.78), in contrast what might be expected from combining individual effects of functional difficulty (PR: 13.92, 95% CI: 9.11 to 21.27) in the younger age group or age among children without functional difficulties (PR: 6.48, 95% CI: 5.57 to 7.55), and suggests a synergistic interaction of older age and functional difficulty on school enrolment, though the confidence interval included 0. There was no evidence of modification of the effect of functional difficulty on non-enrolment by gender on either the multiplicative or the additive scales.

Patterns of modification of the effect of functional difficulty on school non-enrolment by socioeconomic status, education level of the head of household, and site, were similar. The proportion of children without functional difficulty who were not enrolled in school was higher in poorer households, those with a head of household with no formal education, or living in Jawadhu Hills, relative to children in less poor households, with a head of household reporting any formal education, or living in Timiri (PR: 3.36, 95% CI: 3.03, 3.71; PR: 3.52, 95% CI: 3.20, 3.87; PR: 3.47, 95% CI: 3.15, 3.82), respectively. Among children with functional difficulty, the prevalence of non-enrolment was similar across socioeconomic quintiles, head of household education levels, and site. For these factors, on the multiplicative scale, the pattern is shown as the effect of functional difficulty among children in lower socioeconomic conditions, in families with less educated heads of household, or in Jawadhu Hills was of smaller magnitude than that observed among children in less poor or more educated households or those living Timiri. On the additive scale, the results were suggestive of an antagonistic relationship of functional difficulty with each of these factors, particularly education and site, but again confidence intervals included 0.

## Discussion

This large-scale census of children in two sites in Tamil Nadu found that 1.0% of children aged 5–17 years were parent-reported to have disabilities, as assessed through the UNICEF-Washington Group Child Module, and prevalence of functional difficulty was similar between boys and girls. The most prevalent reported functional impairments were being understood and walking. Sensory difficulties (seeing/hearing) or mental health concerns were less commonly reported, which may be because these are more difficult for a parent to recognise in their child. Childhood disability was largely un-related to socio-demographic features but was more prevalent in the Jawadhu Hills compared to Timiri. Children with disabilities were substantially less likely to be enrolled in school compared to children without disabilities (56.9% versus 90.5%). Across the cohort, school exclusion was most prevalent among children with disabilities and 12 + years old. However, the effect of functional difficulty on school exclusion was four times greater among younger children relative to older ones. There was no evidence of heterogeneity in the effect of functional difficulty by gender.

The prevalence of childhood disability of 1.0% reported in this study was lower than estimates from the World Report on Disability (5%) [3] or the Global Burden of Disease (11%) [32], as well as the 2011 India census estimates (1.5% of children aged 5–9 years and 1.8% of children 10–19 years)[17], which is already likely to provide an under-estimate of the true prevalence of disability [18]. These estimates covered children below 5, which may explain some of the discrepancy. It is higher than the India Health and Development Survey (2005), which found a prevalence of 0.37% in children 5–17 years using the Washington Group Short Set (which contains six functional domains rather than the 13 assessed in this study with the Washington-UNICEF Child Functioning Module [33]. A survey in Andhra Pradesh including 1,383 children aged 0–17 years used modules from the UNICEF-Washington Group module to obtain an estimated prevalence of childhood disability of 2.3% (1.4–3.7%) [26, 34]. Measures of behavioural difficulties (e.g. controlling behaviour, play) and mental health (worry) were not included in the Andhra Pradesh survey, which would have pushed the prevalence higher. The Andhra Pradesh survey also used clinical measures to estimate the prevalence of vision, hearing and/or physical impairment or epilepsy, which together affected 2.9% (2.1–4.0%) of children aged 0–17 years. In comparison, our low prevalence might be explained by subjective parent report in comparatively low-and-middle-income settings. Parental perceptions and understanding, their stage of acceptance of disability, and cultural norms can affect parent reporting of symptoms as evidenced from an Indian study [35]. Surveys from five geographically diverse populations in India have also reported higher prevalence than in the current study. A survey of 3,964 children aged 2–9 years found that 9.2% of children 2-<6 years and 13.6% of children 6–9 years had one of seven neurodevelopmental disorders (vison impairment, epilepsy, neuromotor impairments, hearing impairment, speech and language disorders, autism spectrum disorders, and/or intellectual disability) [19]. The higher prevalence of neurodevelopmental disorders might be due to additional screening and specific questionnaires used in that study. Consistent with our findings, there was no gender difference or socio-economic correlates of these conditions.

Other studies support our finding of the lower levels of school inclusion among children with disabilities compared to those without disabilities, including the 2011 census (school attendance 61% of children with disabilities and 71% of all children) [17] and the 2015 Andhra Pradesh study (51% versus 91%) [26, 34]. Similar to our finding that older children with disability are the most frequently non-enrolled in school, increased enrolment of children with disabilities in primary versus higher school levels were also shown in the census, [17] official government enrolment numbers [23], a 2018 national survey [36], and a case-control study in New Delhi [37]. However, our analysis allowed us to observe considerable heterogeneity in the impact of functional difficulty on school enrolment between age strata. Specifically, while all children with functional difficulty were less likely to be enrolled than children without disability, the effect of disability on school inclusion was most pronounced in the younger age group, demonstrating how disability can detrimentally impact the life course at an early stage, because educational exclusion will likely persist. In turn, we show this persistence by estimating the prevalence of non-enrolment to be highest among older children with functional difficulty.

Our examination of modification of the effect of functional difficulty on school non-enrolment highlights the importance of examining modification of effects on both multiplicative and additive scales [29]. For age, for example, it was important to show how much stronger the association between disability and non-enrolment is among younger children, but while the children most at risk of non-enrolment were older children with disabilities. Our analysis of the interaction between socioeconomic difficulty, disability, and school enrolment, suggest that there might be competing risks to non-enrolment. For children with disabilities from poorer backgrounds, although less likely to be in school than their better-off peers, their enrolment was less likely to be affected by their disability *per se*. However, while this is clear on the multiplicative scale, the non-statistically significant interaction on the additive scale warns against over-interpreting the policy implications.

Our current analysis shows that, though there is a considerable difference in enrolment between genders overall, there was no difference in the effect of functional difficulty on school enrolment of children based on gender. The 2018 survey and official enrolment numbers showed slightly higher enrolment of boys with disabilities compared to girls across most levels of schooling [36], while the New Delhi case-control study reported that primary school enrolment was more common in girls with disabilities compared to boys [38]. Household head education and socio-economic status appeared less relevant as correlates of school enrolment among children with disabilities in the New Delhi study [38]. In India and other settings, children with severe disabilities are less likely to be enrolled in school [39]. To address this gap, the Government of India has introduced the Sarva Sikhsha Abhiyan (SSA) where all children irrespective of disabilities can attend school education services [40]. Additional vulnerabilities as highlighted in this study should be addressed to achieve an optimum implementation.

In terms of strengths, this was a large study including 29,044 children identified through an exhaustive census activity. Disability was assessed using the UNICEF-Washington Group Child Functioning Module. Two contrasting sites were included – one rural and one tribal – allowing comparison of findings.

There are also important limitations to consider. The functional difficulty measure utilised caregiver reporting of difficulties, and reported disability may be subjective and not fully captured. Caregiver expectations of childhood functioning may be gendered, biasing comparisons between boys and girls. The educational measure was report-based and focussed on current school enrolment only and did not assess other important aspects of schooling, such as quality of education, educational attainment, social inclusion and freedom from violence and bullying. The World Report on Disability found that when children with disabilities did enrol in school, their dropout rates were higher and they were on average at a lower level of schooling for their age [3], and quality of schooling may be worse for children with disabilities [41]. Studies from India have also highlighted that although progress has been made, gaps in the provision of inclusive education remain, including in teacher training and provision of appropriate and adequate resources [42, 43]. Moreover, it is also known that children with disabilities often experience difficulties at school, such as being more likely to experience violence, whether physical, psychological, or sexual [44], and these measures were not captured in the study. Further, the correlates of educational enrolment used focussed on individual characteristics of the child (e.g. age, gender, household wealth) rather than features of the environment or school (e.g. accessible facilities, staff trained about disability, inclusive societal attitudes).

### Implications for policy, practice, and research

This study demonstrated that the UNICEF-Washington Group Child Functioning Module could be implemented on a large-scale. It produced a relatively conservative estimate of disability, compared to previous reports in India, and so arguably may provide assessment of more severe functional difficulties, although further research is needed in other parts of the country to validate and to explore this issue. The individual questions on functioning allowed us to estimate prevalence in this context with minimal variation. It may be helpful to reduce the full number of questions to allow implementation in more time-constrained activities (e.g. census).

This evidence shows nearly half of children with disabilities are not enrolled in school in this area of Tamil Nadu, despite strong policy commitments made in India towards disability-inclusive education. Monitoring of inclusion therefore remains important, to assess whether these policies are being realised. This exclusion of children with disabilities from education is a violation of their rights. Moreover, education is important for all children, including those with disabilities in terms of improving future job opportunities and earnings, developing friendships and participating in society, and in low resource settings, access to school-based health and nutrition programmes [45]. Efforts are therefore needed to improve school enrolment of children with disabilities, although the evidence base on which interventions are effective to achieve this goal is currently limited [14, 15]. Attending school alone is insufficient, and further investigation is needed to assess the quality of the experience and educational outcomes for children with disabilities, to identify areas where further improvements are needed (e.g. accessibility of facilitates, expertise of teachers, resource allocation) [46]. Again, evidence is lacking on which interventions are effective at improving these broader educational outcomes for children with disabilities in LMICs [14, 15]. The limited data available focusses mostly on interventions that produce individual-level change (e.g. computer skills training of children with disabilities) rather than school/societal interventions (e.g. teacher training on disability, introduction of disability-inclusive educational policies) and so these large evidence gaps need to be addressed with high-quality research.

## Conclusions

At least one in a hundred children in southern India have severe functional difficulties. Among these children with disabilities, nearly half do not attend school. Urgent action is needed to improve educational inclusion of children with disabilities in southern India, and to ensure that they have a quality and safe school experience.

## Figures and Tables

**Table 1:** Occurrence of any functional difficulty and difficulty within functional domains by gender among N = 29,044 children in Tamil Nadu, India

	Male (N = 15,073)	Female (N = 13,971)	All (N = 29,044)
Functional Difficulty	n (%)	n (%)	n (%)
**Any**	149 (1.0)	150 (1.1)	299 (1.0)
**Seeing**	16 (0.1)	18 (0.1)	34 (0.1)
**Hearing**	16 (0.1)	27 (0.2)	43 (0.1)
**Walking**	55 (0.4)	55 (0.4)	110 (0.4)
**Self-Care**	55 (0.4)	44 (0.3)	99 (0.3)
**Being Understood**	68 (0.5)	61 (0.4)	129 (0.4)
**Learning**	51 (0.3)	49 (0.4)	100 (0.3)
**Remembering**	48 (0.3)	44 (0.3)	92 (0.3)
**Concentrating**	43 (0.3)	41 (0.3)	84 (0.3)
**Accepting Change**	40 (0.3)	33 (0.2)	73 (0.3)
**Controlling Behaviour**	45 (0.3)	40 (0.3)	85 (0.3)
**Making Friends**	41 (0.3)	42 (0.3)	83 (0.3)
**Anxiety**	22 (0.1)	20 (0.1)	42 (0.1)
**Depression**	14 (0.1)	15 (0.1)	29 (0.1)

**Table 2:** Association of functional difficulty with factors for the purposes of targeting and understanding clustering of disadvantage among N = 29,044 children in Tamil Nadu, India

	Overall	Functional Difficulty	Crude	Adjusted*
Variable	n (%)	n (%)	PR (95% CI)	PR (95% CI)
**Age Category (years)**				
5–9	10,692 (36.8)	97 (0.9)	REF	REF
10–13	8,864 (30.5)	102 (1.2)	1.27 (0.97, 1.67)	1.27 (0.97, 1.67)
14–17	9,488 (32.7)	100 (1.1)	1.16 (0.88, 1.54)	1.17 (0.88, 1.55)
**Gender**				
Male	15,073 (51.9)	149 (1.0)	REF	REF
Female	13,971 (48.1)	150 (1.1)	1.09 (0.87, 1.36)	1.09 (0.87, 1.37)
**School Enrolment**				
Not in education	2,860 (9.8)	129 (4.5)	REF	REF
Anganwadi/Kindergarten	444 (1.5)	2 (0.5)	0.10 (0.02, 0.40)	0.04 (0.01, 0.19)
Primary school	10,627 (36.6)	73 (0.7)	0.15 (0.11, 0.20)	0.07 (0.04, 0.12)
Middle school	6,551 (22.6)	58 (0.9)	0.20 (0.14, 0.27)	0.11 (0.06, 0.17)
Secondary school	4,288 (14.8)	28 (0.7)	0.14 (0.10, 0.22)	0.12 (0.07, 0.19)
Higher secondary school	3,170 (10.9)	9 (0.3)	0.06 (0.03, 0.12)	0.07 (0.03, 0.14)
College/Diploma/ITI/University	1,104 (3.8)	0 (0.0)	0.00 (0.00, 0.00)	0.00 (0.00, 0.00)
**Household Size**				
<5	11,320 (39.0)	119 (1.1)	REF	REF
≥5	17,724 (61.0)	180 (1.0)	0.97 (0.76, 1.23)	0.94 (0.74, 1.20)
**Socioeconomic Status**				
Less poor (Upper 2 quintiles)	17,563 (60.5)	158 (0.9)	REF	REF
Poorer (Lower 3 quintiles)	11,481 (39.5)	141 (1.2)	1.37 (1.08, 1.73)	1.30 (0.93, 1.83)
**Residence Time**				
<5 years	3,385 (11.7)	40 (1.2)	REF	REF
6–10 years	4,062 (14.0)	37 (0.9)	0.77 (0.48, 1.24)	0.76 (0.48, 1.23)
11–20 years	6,036 (20.8)	64 (1.1)	0.90 (0.59, 1.36)	0.88 (0.58, 1.33)
>20 years	15,561 (53.6)	158 (1.0)	0.86 (0.60, 1.23)	0.85 (0.59, 1.22)
**HoH Education**				
No education	6,893 (23.7)	88 (1.3)	REF	REF
Any primary	5,609 (19.3)	63 (1.1)	0.88 (0.63, 1.24)	0.94 (0.65, 1.36)
Any middle	6,215 (21.4)	57 (0.9)	0.72 (0.51, 1.02)	0.77 (0.53, 1.11)
Any secondary or higher	10,327 (35.6)	91 (0.9)	0.69 (0.51, 0.93)	0.75 (0.53, 1.06)
**HoH Marital Status**				
Married	26,192 (90.2)	265 (1.0)	REF	REF
Previously or never married	2,852 (9.8)	34 (1.2)	1.18 (0.82, 1.70)	1.18 (0.82, 1.70)
**Caste**				
Scheduled tribes	8,322 (28.7)	96 (1.2)	REF	REF
Scheduled caste	6,463 (22.3)	61 (0.9)	0.82 (0.58, 1.15)	1.79 (0.91, 3.51)
Backward caste	7,409 (25.5)	78 (1.1)	0.91 (0.67, 1.25)	2.01 (1.03, 3.91)
Most backward caste	6,588 (22.7)	63 (1.0)	0.83 (0.59, 1.16)	1.81 (0.92, 3.57)
Higher caste	172 (0.6)	0 (0.0)	0.00 (0.00, 0.00)	0.00 (0.00, 0.00)
Other	90 (0.3)	1 (1.1)	0.96 (0.14, 6.78)	1.49 (0.21, 10.44)
**Hindu**				
No	954 (3.3)	10 (1.0)	REF	REF
Yes	28,090 (96.7)	289 (1.0)	0.98 (0.53, 1.83)	0.94 (0.50, 1.76)
**Tamil Speaker**				
No	840 (2.9)	7 (0.8)	REF	REF
Yes	28,204 (97.1)	292 (1.0)	1.24 (0.59, 2.61)	1.19 (0.57, 2.51)
**Site**				
Timiri	20,750 (71.4)	197 (0.9)	REF	REF
Jawadhu Hills	8,294 (28.6)	102 (1.2)	1.30 (1.01, 1.67)	1.30 (1.01, 1.67)

* Adjusted for age category, gender, and site; PR = Prevalence Ratio; CI = Confidence Interval

**Table 3:** Association of selected factors with reported school non-enrolment among N = 29,044 children in Tamil Nadu, India

	Overall	Not enrolled	Crude	Adjusted*
Variable	n (%)	n (%)	PR (95% CI)	PR (95% CI)
**Age Category (years)**				
5–9	10,692 (36.8)	289 (2.7)	REF	REF
10–13	8,864 (30.5)	407 (4.6)	1.70 (1.47, 1.96)	1.70 (1.48, 1.96)
14–17	9,488 (32.7)	2,164 (22.8)	8.44 (7.46, 9.54)	8.57 (7.60, 9.66)
**Gender**				
Male	15,073 (51.9)	1,649 (10.9)	REF	REF
Female	13,971 (48.1)	1,211 (8.7)	0.79 (0.74, 0.85)	0.84 (0.79, 0.90)
**School Enrolment**				
Not in education	2,860 (9.8)	2,860 (100.0)	REF	REF
Anganwadi/Kindergarten	444 (1.5)	0 (0.0)	0.00 (0.00, 0.00)	0.00 (0.00, 0.00)
Primary school	10,627 (36.6)	0 (0.0)	0.00 (0.00, 0.00)	0.00 (0.00, 0.00)
Middle school	6,551 (22.6)	0 (0.0)	0.00 (0.00, 0.00)	0.00 (0.00, 0.00)
Secondary school	4,288 (14.8)	0 (0.0)	0.00 (0.00, 0.00)	0.00 (0.00, 0.00)
Higher secondary school	3,170 (10.9)	0 (0.0)	0.00 (0.00, 0.00)	0.00 (0.00, 0.00)
College/Diploma/ITI/University	1,104 (3.8)	0 (0.0)	0.00 (0.00, 0.00)	0.00 (0.00, 0.00)
**Household Size**				
<5	11,320 (39.0)	1,126 (9.9)	REF	REF
≥5	17,724 (61.0)	1,734 (9.8)	0.98 (0.91, 1.06)	0.98 (0.92, 1.06)
**Socioeconomic Status**				
Less poor (Upper 2 quintiles)	17,563 (60.5)	920 (5.2)	REF	REF
Poorer (Lower 3 quintiles)	11,481 (39.5)	1,940 (16.9)	3.23 (2.98, 3.50)	2.01 (1.82, 2.22)
**Residence Time**				
<5 years	3,385 (11.7)	252 (7.4)	REF	REF
6–10 years	4,062 (14.0)	333 (8.2)	1.10 (0.93, 1.31)	0.97 (0.83, 1.14)
11–20 years	6,036 (20.8)	591 (9.8)	1.32 (1.12, 1.54)	1.05 (0.91, 1.22)
>20 years	15,561 (53.6)	1,684 (10.8)	1.45 (1.26, 1.68)	1.18 (1.04, 1.35)
**HoH Education**				
No education	6,893 (23.7)	1,464 (21.2)	REF	REF
Any primary	5,609 (19.3)	567 (10.1)	0.48 (0.43, 0.52)	0.69 (0.63, 0.77)
Any middle	6,215 (21.4)	440 (7.1)	0.33 (0.30, 0.37)	0.54 (0.49, 0.60)
Any secondary or higher	10,327 (35.6)	389 (3.8)	0.18 (0.16, 0.20)	0.29 (0.26, 0.33)
**HoH Marital Status**				
Married	26,192 (90.2)	2,457 (9.4)	REF	REF
Previously or never married	2,852 (9.8)	403 (14.1)	1.51 (1.35, 1.68)	1.38 (1.26, 1.52)
**Caste**				
Scheduled tribes	8,322 (28.7)	1,679 (20.2)	REF	REF
Scheduled caste	6,463 (22.3)	510 (7.9)	0.39 (0.35, 0.43)	0.39 (0.31, 0.49)
Backward caste	7,409 (25.5)	309 (4.2)	0.21 (0.18, 0.23)	0.21 (0.16, 0.26)
Most backward caste	6,588 (22.7)	352 (5.3)	0.26 (0.24, 0.30)	0.27 (0.21, 0.34)
Higher caste	172 (0.6)	4 (2.3)	0.12 (0.04, 0.31)	0.11 (0.04, 0.28)
Other	90 (0.3)	6 (6.7)	0.33 (0.15, 0.70)	0.36 (0.17, 0.75)
**Hindu**				
No	954 (3.3)	64 (6.7)	REF	REF
Yes	28,090 (96.7)	2,796 (10.0)	1.48 (1.15, 1.91)	1.13 (0.88, 1.45)
**Tamil Speaker**				
No	840 (2.9)	71 (8.5)	REF	REF
Yes	28,204 (97.1)	2,789 (9.9)	1.17 (0.92, 1.49)	0.92 (0.73, 1.16)
**Site**				
Timiri	20,750 (71.4)	1,232 (5.9)	REF	REF
Jawadhu Hills	8,294 (28.6)	1,628 (19.6)	3.31 (3.06, 3.57)	3.38 (3.14, 3.63)

* Adjusted for age category, gender, and site; PR = Prevalence Ratio; CI = Confidence Interval

**Table 4:** Modification of the effect of functional difficulty on school non-enrolment by selected sociodemographic factors among N = 29,044 children in Tamil Nadu, India, adjusted for age, gender, and site.

	No Functional Difficulty		Any Functional Difficulty		Effect of FD	EM Measures	
	Non-enrolment n/N (%)	PR (95% CI)	Non-enrolment n/N (%)	PR (95% CI)	PR (95% CI)	Multiplicative (95% CI)	Additive (95% CI)
Overall (crude)	2,731/28,745 (10)	1	129/299 (43)	4.54 (3.95, 5.21)			
Overall (adjusted)*	2,731/28,745 (10)	1	129/299 (43)	4.59 (3.87, 5.43)			
**Age Category (years)**						0.27 (0.17, 0.43)	3.74 (−3.33, 10.75)
5–11	295/12,643 (2.3)	1	38/117 (32.5)	12.06 (8.14, 17.87)	12.06 (8.14, 17.87)		
12–17	2,436/16,102 (15)	6.49 (5.64, 7.47)	91/182 (50)	21.29 (16.20, 27.98)	3.28 (2.57, 4.19)		
**Gender**						1.05 (0.70, 1.59)	0.95 (−0.91, 2.93)
Female	1,148/13,821 (8.3)	1	63/150 (42.0)	4.07 (3.04, 5.46)	4.07 (3.04, 5.46)		
Male	1,583/14,924 (11)	1.22 (1.12, 1.34)	66/149 (44)	5.25 (3.94, 6.99)	4.29 (3.22, 5.70)		
**Socioeconomic Status**						0.40 (0.27, 0.60)	−2.27 (−5.09, 0.39)
Less poor (Upper 2 quintiles)	857/17,405 (4.9)	1	63/158 (39.9)	7.23 (5.39, 9.69)	7.23 (5.39, 9.69)		
Poorer (Lower 3 quintiles)	1,874/11,340 (17)	2.07 (1.83, 2.35)	66/141 (47)	6.03 (4.48, 8.12)	2.91 (2.19, 3.86)		
**No HoH Education**						0.30 (0.20, 0.47)	−3.39 (−5.71, -0.92)
Any education	1,306/21,940 (6.0)	1	90/211 (42.7)	6.78 (5.31, 8.65)	6.78 (5.31, 8.65)		
No education	1,425/6,805 (21)	2.26 (2.05, 2.50)	39/88 (44)	4.65 (3.22, 6.70)	2.05 (1.43, 2.95)		
**Site**						0.33 (0.21, 0.50)	−1.57 (−4.64, 2.07)
Timiri	1,146/20,553 (5.6)	1	86/197 (43.7)	7.06 (5.47, 9.10)	7.06 (5.47, 9.10)		
Jawadhu Hills	1,585/8,192 (19)	3.46 (3.17, 3.78)	43/102 (42)	7.95 (5.58, 11.31)	2.30 (1.62, 3.26)		

* Adjusted for age category, gender, and site; PR = Prevalence Ratio; CI = Confidence Interval; FD = Functional Difficulty; EM = Effect Modification

## Data Availability

The data that support the findings have been archived, but restrictions apply to the availability of these data, and so have not been made publicly available. The data may be made available from the authors upon reasonable request after due oversight from an institutional ethical review process.

## Highlights

- At least one in a hundred children have severe functional difficulties in this population in Tamil Nadu, India
- Functional difficulty was a strong predictor of school non-enrolment, independent of age, gender, and sub-site
- Rates of school inclusion for children with functional difficulties differed between age and socioeconomic groups

## List of abbreviations

CFM: Child Functioning Module
CI: Confidence Interval
HUD: Health Unit District
MDA: Mass Drug Administration
PCA: Principal Component Analysis
PR: Prevalence Ratio
RERI: Relative Excess Risk due to Interaction
RTE: Right to Educate
SES: Socioeconomic Status
STH: Soil-transmitted Helminths
UN: United Nations
UNESCO: United Nations Educational, Scientific and Cultural Organization
UNICEF: United Nations Children’s Fund
